# Comparative Study of Snodgrass and Mathieu's Procedure for Primary Hypospadias Repair

**DOI:** 10.1155/2014/249765

**Published:** 2014-04-27

**Authors:** Raashid Hamid, Aejaz A. Baba, Altaf H. Shera

**Affiliations:** Department of Pediatric and Neonatal Surgery, SKIMS, Srinagar, Jammu and Kashmir 190011, India

## Abstract

*Objective*. Present study was undertaken to compare the results of two single stage hypospadias repairs, namely, Tubularized Incised Plate (TIP) repair and Mathieu's repair. *Methods*. The study included 100 patients of distal penile hypospadias, from January, 2008 to January, 2013. After a detailed history, local examination was performed with reference to the site of meatus, shape of glans, and presence of chordee. TIP repair was performed in 52 patients and Mathieu's repair in 48 patients. On follow-up, the patients were examined for the position of meatus, shape of meatus, urinary stream, urethrocutaneous fistula, and stricture formation. *Results*. The mean age of presentation was 6.2 ± 3.2 years (range 1.5–15years). The mean operative time was 63.7 ± 14.3 (45–90) minutes and 95.0 ± 19.1 (70–125) minutes in TIP and Mathieu's repair, respectively.Complications after surgery were urethero cutaneous fistula in 3(5.76%) and 7 (14.5%), meatal stenosis in 3(5.33%) and 4(8.33%), wound infection in 19.2% and 8.3% cases in TIP repair and Mathieu repair, respectively. The shape of meatus was slit-like and vertically oriented in 48(92.3%) patients who had undergone TIP repair. *Conclusion*. The Snodgrass repair is significantly faster, with more natural cosmetic appearance of the meatus than the Mathieu's repair.

## 1. Introduction

Procedures established to correct distal penile hypospadias are the tubularized incised plate urethroplasty (TIP repair), Thiersch-Duplay, Mathieu's repair, Mustarde, and MAGPI. Among these procedures TIP and Mathieu's urethroplasty have been widely practiced. Mathieu's perimeatal based flap repair was first described in 1932; it is usually applied for correcting coronal and subcoronal defects [1]. Furthermore, Mathieu's repair creates a horizontal and rounded meatus which is cosmetically less acceptable than a normal vertical slit-like meatus. Therefore, TIP urethroplasty is a technique with a low complication rate for correcting distal hypospadias by tubularizing the incised urethral plate to create a vertical meatus [2]. In both Mathieu's and tubularized incised plate urethroplasty, the urethral plate is used which makes the postrepair appearance regarding glans shape and external meatus near to natural [3]. The choice between the procedures depends more on surgeon's experience rather than scientific evidence. The study aims to compare the results of these two methods of hypospadias repairs.

## 2. Material and Methods

**Figure 1:**
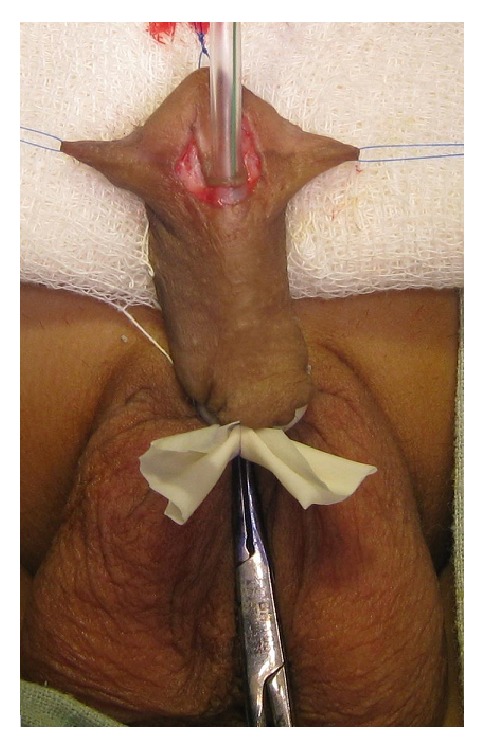
Perimeatal based “U” shaped incision.

**Figure 2:**
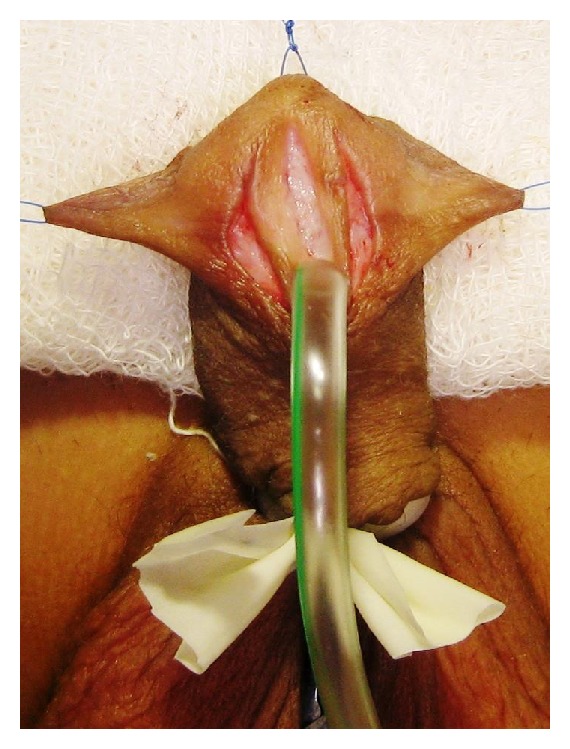
Tubularized incised plate urethroplasty.

**Figure 3:**
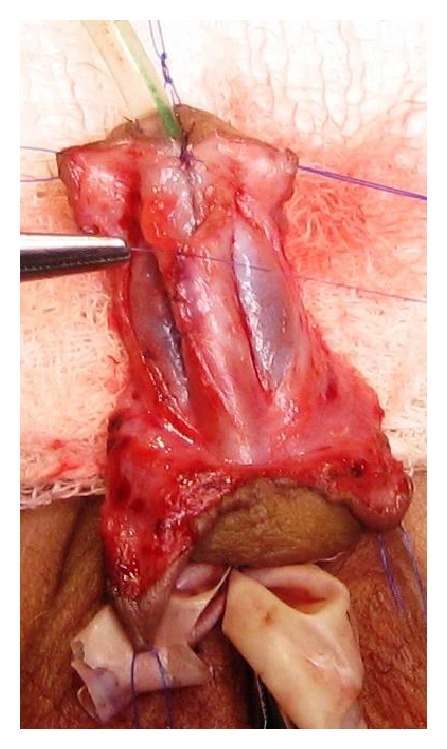
Formation of neourethra.

**Figure 4:**
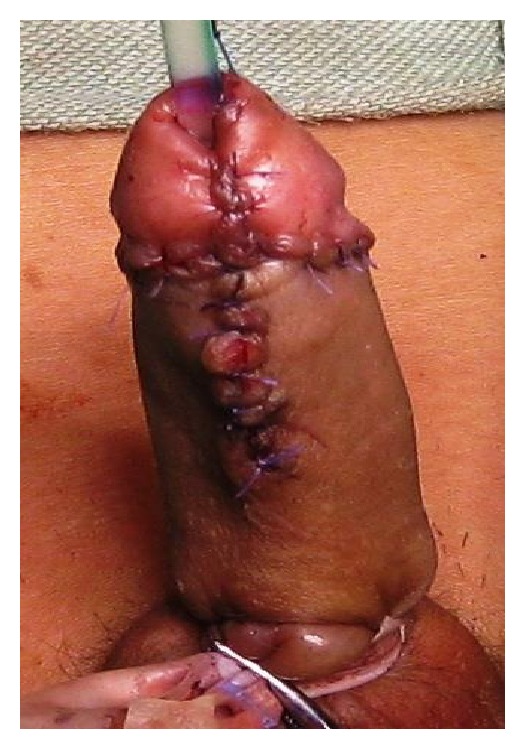
Snodgrass procedure completed.

This was a prospective study between January 2008 and January 2013. A total of 100 patients with primary distal hypospadias were included in the study. These patients were randomly assigned as group-A with 52 patients in whom TIP repair was accomplished and group-B having 48 patients in whom Mathieu's repair was performed. The previously operated cases of distal penile hypospadias with extensive scarring, severe chordee likely to necessitate a staged procedure, and patients with micropenis with poorly developed urethral plate and urethral groove were excluded from the study. Repair of the hypospadias was done under general anesthesia and endotracheal intubation. In TIP repair, a segment of the urethral plate 8–10 mm wide was marked out distal to urethral meatus and incision was made along the lateral borders of the urethral plate. An incision was made subcoronally and was completed ventrally as “U” shape proximal to urethral meatus. After carefully degloving the penis, glanular wings were adequately developed lateral to the urethral plate. A midline vertical incision was made with a knife in the urethral plate. The neourethra was reconstructed around 6 or 8 Fr catheter using multiple interrupted sutures with polygalactin 6-0 with knots outside. Glanuloplasty was performed with 6-0 polygalactin in two layers. Meatoplasty and skin cover were completed (Figures 1, 2, 3, and 4).

In Mathieu's repair, the urethral plate and perimeatal based flap were marked. Typically, the width of 7.5 mm was measured for the proximal flap. Longitudinal lines outlining the urethral plate were then drawn extending along the length of required flap. Urethral plate and proximal shaft skin were incised about 7-8 mm wide and the penis was degloved after subcoronal circumferential incision. Granular wings were developed by deep dissection under glans to perform tension free glanuloplasty. Proximal shaft skin flap was mobilized and transposed toward the urethral plate. This flap was folded over the urethral meatus and neourethra formed over 6 or 8 Fr tube wit interrupted 6-0 polygalactin knots outside. Glanuloplasty and skin coverage completed the procedure.

In both the groups chordee was managed by degloving of penis, and some patients needed dorsal plication also to address the residual chordee after degloving.

**Figure 5:**
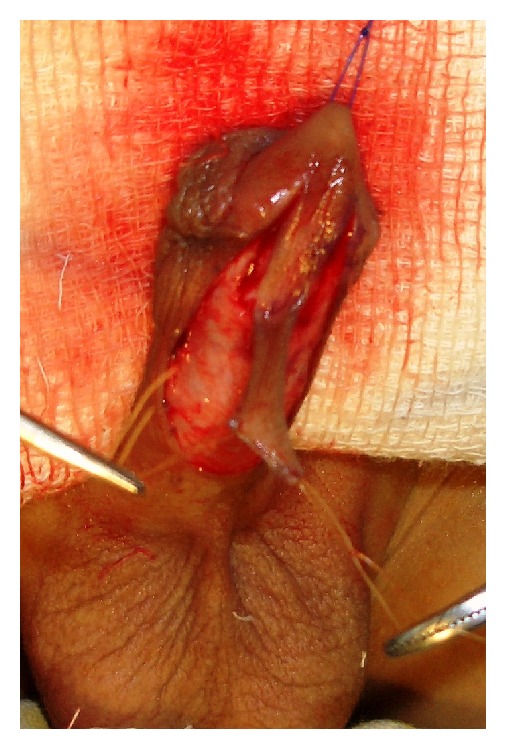
Perimeatal based flap in Mathieu's procedure.

**Figure 6:**
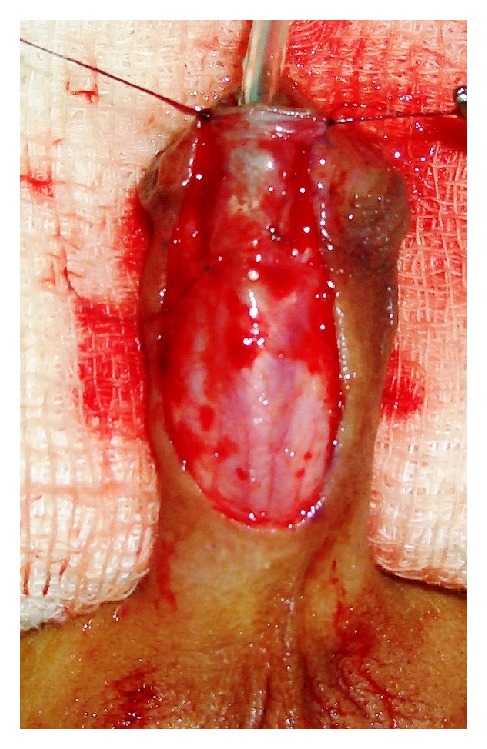
Perimeatal based flap applied to form neourethra.

**Figure 7:**
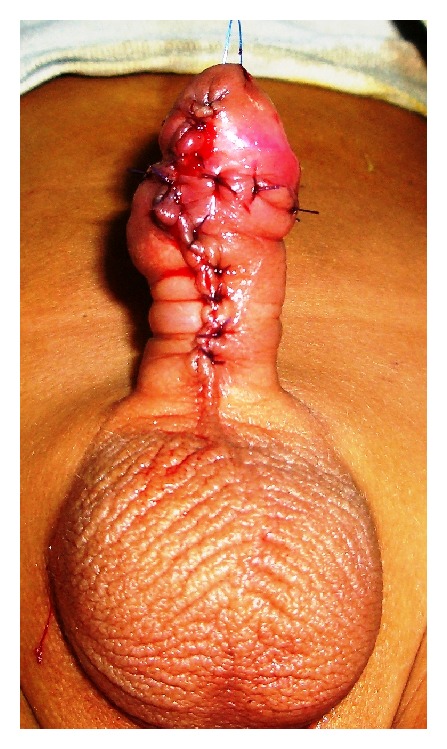
Mathieu's procedure completed.

Dressing of the penis was first opened 5 days after surgery, and the wound was laid open. The catheter was removed 7 to 14 days postoperatively; patients were discharged home. Patients were examined in follow-up weekly for the first month, four weekly for next 6 months, 3 monthly for next year, and thereafter 6 monthly. Impression about the cosmetic outcome was made by a surgeon who was unaware about the type of procedure performed (TIP or Mathieu's repair) to minimize the observer's bias. The mean follow-up of the patients was 17 months, ranging from 1 to 44 months in both the groups (Figures 5, 6, and 7).

## 3. Results

The mean age in the group-A was 6.4 ± 3.3 (2.5–15) years and in group-B it was 5.9 ± 3.1 (1.5–14) years. The difference in the age groups was statistically nonsignificant between the two groups.

Sixteen (30.76%) patients in group-A had coronal, 14 (26.92%) had subcoronal, and 22 (42.40%) had distal penile hypospadias while in group-B 24 (50.00%) patients had coronal, 8 (16.66%) had subcoronal, and 6 (33.33%) had distal penile hypospadias. The difference was statistically insignificant between the two groups (Table 1). The mean operative time in TIP repair (group-A) was 63.7 ± 14.4 (45–90) minutes and in Mathieu's repair (group-B) was 95.0 ± 19.1 (70–125) minutes. The difference in the operative time between the two surgical procedures was statistically significant (*P* < 0.05). Meatal stenosis developed in 3 (5.76%) and 4 (8.33%) patients in group-A and group-B, respectively, meatal stenosis was managed by dilatation and one patient from each group needed meatoplasty. Urethrocutaneous fistula developed in 3 (5.76%) patients of group-A and 6 (12.5%) patients in group-B. Wound infection developed in 3 (5.76%) and 4 (8.33%) patients in group-A and group-B, respectively, and was managed by antibiotics (Table 2).

On follow-up the cosmetic appearance of the penis was excellent in all the patients (100%) in group-A; in group-B excellent cosmesis was achieved in 20 (41.66%) patients. In group-B 8 (16.7%) patients had mild torsion of the shaft. This difference was statistically significant between the two groups (*P* < 0.05). The shape of meatus was slit like and vertically oriented in 48 (92.3%) patients who had undergone TIP repair.

The shape of meatus was round and regular in 44 (91.6%) patients who had undergone Mathieu's repair.

## 4. Discussion

The ultimate goal of any hypospadias repair is to achieve a functionally and cosmetically normal penis. Even in the hands of most experienced surgeons, hypospadias repair is associated with a number of complications ranging from urethrocutaneous fistula to the complete breakdown of neourethra requiring total reconstruction of the different single stage hypospadias repairs. The tubularized incised plate urethroplasty and Mathieu′s repair have been widely practiced. Final cosmetic results and normal penile functioning are two important considerations in hypospadias surgery.

The mean age (6.4 ± 3.3 years) of the patients in our study was more as compared to the age reported by other authors [4–6]. This may be due to lack of awareness and education on the part of parents and delayed referral from rural health care centres. The position of meatus in our study groups A and B was consistent with that reported by Anwar-ul-haq et al., Bath et al., and Sugarman et al. [6–8].

In our series, chordee was seen in 26 (48%) patients in group-A and 22 (45.83%) in group-B. Incidence of chordee was different in different series; it was 35% in Sweet et al. series, 100% in Tonvichien and Niramis case series, and 19.9% in Singh et al. series of cases [9–11]. At a mean age of 4 years plateau uroflow curve (versus normal bell curve) was observed in 17 (32.69%) patients who underwent TIP urethroplasty and 14 out of 18 (29.16%) who underwent Mathieu's repair. The meatal based flap is commonly used urethroplasty for distal penile hypospadias; the reported complication of Mathieu's urethroplast is between 5 and 21% for distal penile hypospadias. There is also an increased risk of meatal stenosis because there is reduced blood flow to the distal part of the flap [5]. Other potential problems of perimeatal based flap are hair growth at meatus and cosmetically undesirable horizontal and rounded meatus. The concept of incising the urethral plate enables tubularization and creating a neourethral tube irrespective of the glans configuration or the location of the meatus [12]. This deep incision does not compromise its viability and reepithelialization of neourethra as compared to Mathieu's in which two suture lines are needed on two sides. In TIP repair, only one suture line is used in creating a neourethral tube. TIP repair was done in significantly shorter time than Mathieu's repair which has also been described by Oswald et al. and Guo et al. [12, 13].

In our series complications were seen in 28 (28.0%) patients. The complications included wound infection seen in 3 (5.76%) patients in the group-A and 4 (8.3%) patients in the group-B. Complications do occur after every hypospadias repair. Currently there is expected complication rate of 5–10% mostly fistulas in one stage urethroplasty [14] (Table 3).

In our series excellent cosmesis was achieved in 52 (100%) patients in the group-A and in the group-B cosmetic appearance was excellent in 40 (83.3%) patients and 8 (16.7%) patients had mild torsion of shaft. Our cosmetic outcome after TIP urethroplasty was similar to that reported by authors like Anwar-ul-haq et al., Guo et al., and Imamoğlu and Bakirtaş [6, 13, 15].

As evident in our series, the urethrocutaneous fistula and meatal stenosis rate was more in Mathieu's group as compared to that of Snodgrass group. One explanation for this is the need for two suture lines in case of onlay flap technique on either side which might be jeopardizing the vascular supply of the flap. Although the expected complication rate in Mathieu's urethroplasty ranges between 5 and 21% for distal repair, in group-A the incidence of UCF was 5.76% only which was significantly lower than what was observed after Mathieu's urethroplasty. Cosmesis of penis is of concern to the parents which was best achieved with TIP urethroplasty as compared to Mathieu's urethroplasty. TIP creates a vertically oriented, slit like normal looking meatus which is cosmetically desirable.

## 5. Conclusion

Our study has revealed that TIP urethroplasty has an edge on Mathieu's urethroplasty, so we recommend the TIP urethroplasty in all primary and distal cases of hypospadias. TIP repair is associated with excellent cosmesis and few manageable complications. It offers a safe and reliable modality for primary repair of distal penile hypospadias. Cosmetic appearance of the external urethral meatus is highly satisfactory with tubularized incised plate urethroplasty.

## Figures and Tables

**Table 1:** Patient Characteristics.

	TIP repair (group-A) (*n* = 52)	Mathieu's (group-B) (*n* = 48)	*P* value
Age ( Years)	6.3 ± 3.3	5.9 ± 3.1	0.317

Position of meatus	Coronal—16 (30.76%)	24 (50.00%)	0.005*
	Subcoronal—14 (26.92%)	8 (16.66%)	
	Distal—22 (42.30%)	6 (33.33%)	

Circumcised	10 (19.23%)	11 (22.91%)	0.807

Chordee	25 (48.07%)	22 (45.83%)	0.844

*Significant at 5%.

**Table 2:** Operative time and complications.

	TIP repair (group-A) (*n* = 52)	Mathieu's (group-B) (*n* = 48)	*P* value
Operative time	63.7 ± 14.4	95 ± 9.1	0.003*

Complications	Snodgrass (group-A) (*n* = 52)	Mathieu's (group-B) (*n* = 48)	*P* value

Wound infection	3 (5.76%)	4 (8.33%)	0.153
Bladder spasms	10 (19.83%)	9 (18.75%)	1.000
Flap necrosis	0	3 (6.25%)	0.107
Meatal stenosis	3 (5.76%)	4 (8.33%)	0.707
Urethrocutaneous Fistula	3 (5.76%)	6 (12.5%)	0.305
Plateau uroflow curve	17 (32.69%)	14 (29.16%)	0.829

*Significant at 5%.

**Table 3:** Literature review.

Variable	References	Snodgrass	Mathieu
Mean operative time (in minutes)	Oswald et al. [12]	75 min.	115 min.
	Guo et al. [13]	106.1 min.	94.0 min.
	Chatterjee et al. [14]	45–110 min.	40–80 min.
	**Our series**	**63.7 min.**	**95.0 min**

Postoperative complications (1) Urethrocutaneous fistula	Guo et al. [13]	8.3%	25.6%
	Guo et al. [13]	13.3%	5.5%
	Oswald et al. [12]	0%	6.6%
	Chatterjee et al. [14]	3.3%	7.7%
	Imamoğlu and Bakirtaş [15]	7.1%	7.4%
	Tonvichien and Niramis [10]	14.0%	—
	Singh et al. [11]	11.5%	—
	**Our series**	**5.76%**	**12.5%**
(2) Meatal stenosis	Chatterjee et al. [14]	5.5%	5.5%
	Guo et al. [13]	6.6%	0%
	Oswald et al. [12]	0%	3.3%
	Al-Saied and Gamal [3]	6.2%	—
	Singh et al. [11]	1.9%	—
	**Our series**	**5.76%**	**12.5%**
(3) Repair breakdown	Chatterjee et al. [14]	1.1%	1.1%
	Guo et al. [13]	8.3%	2.3%
	Imamoğlu and Bakirtaş [15]	5.3%	7.4%
	Singh et al. [11]	1.9%	—
	Guo et al. [13]	0%	0%
	**Our series**	**0%**	**6.25%**

Mean hospital stay (in days)	Imamoğlu and Bakirtaş [15]	7.5 days	5.7 days
	Guo et al. [13]	4.5 days	3.9 days
	Chatterjee et al. [14]	2.5 days	3.0 days
	**Our series**	**15.4 days**	**14.8 days**

Shape of meatus	Oswald et al. [12]	Slit like in 100%	Rounded in 100%
	**Our series**	**Slit like in 92.3%**	**Rounded in 100%**

Cosmesis	Chatterjee et al. [14]	Excellent
	Guo et al. [13]	Highly satisfactory
	Imamoğlu and Bakirtaş [15]	Highly satisfactory
	**Our series**	**Excellent in 100%**	**Excellent in 83.3%**
